# Challenges and perspectives in analyzing health in the Brazilian Amazon: a look at population-based studies

**DOI:** 10.1590/0102-311XEN045824

**Published:** 2025-03-24

**Authors:** Mônica da Silva-Nunes, Ana Paula Dal’Asta, Cláudia Torres Codeço

**Affiliations:** 1 Universidade Federal de São Carlos, São Carlos, Brasil.; 2 Universidade Federal do Acre, Rio Branco, Brasil.; 3 Instituto Nacional de Pesquisas Espaciais, São José dos Campos, Brasil.; 4 Programa de Computação Científica, Fundação Oswaldo Cruz, Rio de Janeiro, Brasil.

The Brazilian Amazon has great environmental and social heterogeneity, and is home to several ecosystems vital for production and maintenance of biodiversity, of the water cycle, and of other ecosystem services ^1^. The population occupation and dispersion pattern throughout the Amazon resulted in a myriad of human settlements interconnected by rivers and land, which was modified by the incorporation of intervention processes in the territory. From 1970 to 2020 ^1^, the region saw its population increase from 8 to 28 million with profound changes in the socio-spatial distribution and urbanization pattern, forming the so-called urbanized forest ^2^. In this environment, ways of life and productive systems persist and resist for which the forest is a resource and a means of reproduction of life, in a complex space of relations and interactions with the new employer production systems, in which the forest is only an input ^3^.

The socio-environmental diversity and specificity in the Amazon poses challenges for scientific knowledge production ^3^. Observations of health and disease processes and their determinants is heterogeneous in their quality and representativeness of the populations studied, both human and nonhuman ^4^. This is evident in biodiversity studies that show a concentration of surveys in areas of easier access and close to research institutions ^5^. Among epidemiological studies, clinical studies with patients recruited in care services, usually residents of state capitals that house academic institutions, predominate. Population-based studies in the form of local surveys are less frequent ^6^ ^,^ ^7^ ^,^ ^8^. Apart from urban centers, there are few examples of research along a gradient of urban-rural or riverine landscapes ^9^.

Based on the authors’ experience in field research in the region, this study analyzes the specificities of health and disease research in the Amazon, with emphasis on population-based studies, addressing methodological aspects to be considered when designing surveys and cohorts in the area.

## Health status in the Amazon based on public-based indicators

Surveying the health status in the Amazon and its determinants results from the analysis of data collected for this specific purpose, such as epidemiological studies, or data from more generic data collection systems, such as the national registration systems ^3^. Overall, studies based on these data sources highlight the lower life expectancy of residents in the Amazon Federative Units compared with the rest of Brazil, as well as differences between these units. When stratified by age group, communicable diseases are the main responsible for mortality in the first five years of life and noncommunicable diseases are responsible for mortality in those over 60 years of age ^3^. Such global analyses can hide factors that are only evident at more detailed scales.

## Population-based epidemiological studies

Analytical population-based studies seek to measure the association between exposure and a health-related condition by conducting cross-sectional surveys, case-control studies, or prospective cohort research. A first challenge in elaborating population-based research in the Amazon is defining the target population. Defining inclusion and exclusion criteria comes up against issues such as the lack of adequate registration of localities at the intra-municipal scale, the inadequate classification of urban or rural areas, the lack of a classification system to identify localities with more ribeirinha or upland characteristics, or to identify socially distinct areas such as *quilombola* remnants. These geographic databases are not consolidated, resulting in inconsistencies that affect the quality of studies.

Population size is another challenge. Designing traditional epidemiological studies requires good population estimates to support sample calculations. If the population is large enough to enable a sample design, generating inferences requires knowing the population at risk. Lack of population data on this scale is common. Data from census tracts may be an option, but sometimes these are not harmonized with socially or ecologically identified territorial bases. Another source is primary care data, such as family health ^6^. People’s mobility also affects population studies, as it is frequent to change housing within the same municipality, or multiple dwellings in rural and urban areas, and this must be considered when collecting data ^6^ ^,^ ^7^. House construction is relatively easy, especially in small municipalities, and the family unit varies greatly, with frequent changes in the dwellers composition of residences ^7^. This can lead to considerable losses from follow-up, and requires devising specific strategies for the study.

Localities with small populations requires conducting a census to obtain only descriptive results. These studies can provide valuable knowledge about certain populations, for example Indigenous peoples of a certain region, or a small ribeirinha population with a traditional lifestyle ^9^ ^,^ ^10^.

The Amazon is home to metropolitan areas in the middle of the forest, urban centers, communities and villages, Indigenous areas, *quilombolas*, farms, peasant arrangements, rubber plantations, artisanal mining, large project camps, agrarian reform settlements, and landless encampments. Seasonality affects accessibility, with better access by road in the dry season and better river access in the rainy season. Conflict situations, environmental disaster, illegal trade in the Amazon territory add another layer of complexity to the health of populations and their accessibility. These spatial and temporal heterogeneities of access can result in observational bias if exposure, for example, correlates with these spatiotemporal factors. Overcoming these biases requires investment in research infrastructure and higher costs, including time. Research in ribeirinha communities tends to require more travel time since residents live more distant from each other. Another obstacle to be overcome is the remote condition of the study populations, often in places without electricity, in which the use of electronic devices for data collection becomes difficult or even impossible. This condition is frequent in ribeirinha or rural populations where there is no energy or only power from generators, with high cost and low predictability.

Finally, each of these locations has its peculiarities regarding access which determines the strategy for selecting participants, the composition of the research team, and the research objectives themselves.

Another challenge for studies in the Amazon region is selecting instruments that are valid for the region, since most tools are validated in populations living in very different environments and living conditions. Cultural diversity, which includes linguistic diversity, poses the need for validation on a local scale via qualitative studies. An example is the adaptation of psychometric scales of food insecurity among vulnerable Indigenous populations ^11^. Another example is socioeconomic indicators. To define income in the Amazon, one must needs consider the significant role of the informal market. Wage labor does not always exist. Income can be extremely variable, differing from month to month, week to week, or day to day. Determining the ownership of consumer goods can be a good strategy when the nominal income is uncertain or does not want to be declared ^6^ ^,^ ^9^, and enables the creation of wealth indices capable of providing stratification by income. However, for some populations monetary income is not associated with their way of life and social reproduction. Considering a variety of indicators to capture situations of deprivation offers a more complete picture of the socioeconomic realities of Amazonian populations. Studies on poverty based on a multidimensional perspective point to the complexity in assessing what it means to be poor in the Amazon and how traditional indicators can mask the real deprivations of the population ^12^.

For example, in several places most households are made of wood and the floor material, the presence of running water inside or outside the household, or ownership of a motor boat, are the determining variables for economic classification ^6^. Various authors ^6^ ^,^ ^7^ ^,^ ^9^ cite as the main descriptors of the socioeconomic conditions of urban and rural populations in the inland Amazon the presence or absence of electricity in the household, the type of floor, the presence of open sewage, the type of sanitary installation (cesspool or toilet with flush), the destination of the garbage (burned or buried) and the origin or treatment of drinking water (mineral water, filtered, boiled or chlorinated well or tap water), the use of firewood as fuel for cooking. In recent decades, the provision of government aid, especially Brazilian Income Transfer Program, has proven to be an indicator for the socioeconomic stratification of the Amazonian population ^7^ ^,^ ^13^.

Another important point is the need to think about alternative ways of identifying people in the absence of formal records, especially in less urbanized areas. For example, nominal and age records tend to vary greatly. It is common for individuals to inform surnames different from those registered in a notary’s office, both because of the informality of the population and for illiteracy reasons. These are evidence of the need for validating instruments in a local context.

## Conclusion

Understanding the different realities that make up this region can only be achieved with this detailed look at the Amazonian particularities enabled via population-based studies. Due to its great extension, environmental heterogeneity, and human occupation, understanding the Amazon and how health processes take place there requires a great multiplicity of studies. It is these studies that can advance beyond the use of secondary databases that often have standardized variables designed for the national territory with limitations for specific contexts, and that can contribute to breaking narratives of invisibilization of populations and processes. Additionally, qualitative studies can also help to analyze and understand health-disease processes among Amazonian populations on different scales than quantitative studies.

Achieving this goal demands increasing research promotion in the Amazon, a strategy that has received greater government emphasis recently, especially by stimulating research in local institutions. Secondly, it requires well-conducted studies with the involvement of people who know these specificities, and with good training in research in the Amazon. This goal can be achieved in the short and medium term by strengthening graduate programs based in Amazonian institutions and by engaging in collaborative networks. These networks need to be additive, that is, allow people’s engagement, and not restrictive (such as establishing numerical prerequisites for submitting research proposals). In the long term, however, this policy must be strengthened with the training of human resources at the most different levels, starting with basic education, and available to all, whether they live in cities, forests, riversides or the countryside. Finally, long-term strategies of at least a decade or more must be draw up.
