# Supplementary material for: Challenges and perspectives in analyzing health in the Brazilian Amazon: a look at population-based studies
Source: Cad Saude Publica. 2025 Mar 24;41(Suppl 1):e00045824. doi: 10.1590/0102-311XEN045824 (PMC11940849; doi:10.1590/0102-311XEN045824)
Supplement: Translation

# Translation: Desafios e perspectivas na análise da saúde na Amazônia brasileira: um olhar sobre estudos de base populacional

**DOI:** 10.1590/0102-311XPT045824

**Authors:** Mônica da Silva-Nunes, Ana Paula Dal’Asta, Cláudia Torres Codeço

**Affiliations:** 1 Universidade Federal de São Carlos, São Carlos, Brasil.; 2 Universidade Federal do Acre, Rio Branco, Brasil.; 3 Instituto Nacional de Pesquisas Espaciais, São José dos Campos, Brasil.; 4 Programa de Computação Científica, Fundação Oswaldo Cruz, Rio de Janeiro, Brasil.

A Amazônia brasileira apresenta grande heterogeneidade ambiental e social e abriga vários ecossistemas vitais para a produção e a manutenção da biodiversidade, do ciclo hídrico e de outros serviços ecossistêmicos ^1^. O padrão de ocupação e dispersão da população pelo território amazônico resultou numa miríade de assentamentos humanos, interligados por rios e terra, que foi se modificando ao incorporar processos de intervenção no território. Entre 1970 e 2020 ^1^, a região observou sua população aumentar de 8 para 28 milhões, com mudanças profundas na distribuição socioespacial e no padrão de urbanização, conformando a chamada floresta urbanizada ^2^. Nesse ambiente, persistem e resistem modos de vida e sistemas produtivos para os quais a floresta é recurso e meio de reprodução da vida, em um espaço complexo de relações e interações com os novos sistemas produtivos patronais, no qual a floresta é apenas insumo ^3^.

O contexto de diversidade e especificidade socioambiental na Amazônia coloca desafios para a produção de conhecimento científico ^3^. A observação dos processos de saúde e doença e seus determinantes é heterogênea na qualidade e representatividade das populações estudadas, tanto humanas como não humanas ^4^. Isso é evidente em estudos de biodiversidade que mostram a concentração de levantamentos em áreas de fácil acesso e perto de instituições de pesquisa ^5^. Entre os estudos epidemiológicos, há predominância de estudos clínicos em pacientes captados no atendimento, em geral residentes de capitais onde há instituições acadêmicas. Estudos de base populacional na forma de inquéritos locais são menos frequentes ^6^ ^,^ ^7^ ^,^ ^8^. Para além dos centros urbanos, são poucos os exemplos de inquéritos realizados ao longo de um gradiente de paisagens urbano-rurais ou ribeirinhas ^9^.

A partir da experiência das autoras em pesquisas de campo na região, o objetivo deste texto foi analisar as especificidades da pesquisa em saúde e doença na Amazônia, com ênfase em estudos de base populacional, abordando aspectos metodológicos a serem considerados no delineamento de inquéritos e coortes na área.

## Situação de saúde na Amazônia a partir de indicadores de bases públicas

O levantamento de situação de saúde na Amazônia e seus determinantes resulta da análise de dados coletados para esse fim específico, como inquéritos epidemiológicos, ou pode ser um excerto de sistemas mais genéricos de coleta, como os sistemas nacionais de registro ^3^. De forma geral, estudos baseados nessas fontes ressaltam a menor expectativa de vida dos residentes de Unidades da Federação da Região Amazônica em comparação com o resto do país, além das diferenças entre essas unidades. Quando estratificadas por faixa etária, as doenças transmissíveis são as principais responsáveis pela mortalidade nos primeiros cinco anos de vida e as doenças não transmissíveis responsáveis pela mortalidade em pessoas com mais de 60 anos ^3^. Análises nessa escala podem esconder fatores que só são evidentes em escalas mais detalhadas.

## Estudos epidemiológicos de base populacional

Estudos de base populacional de natureza analítica visam mensurar a associação entre uma exposição e uma condição relacionada à saúde por meio da realização de inquéritos seccionais, estudos de caso-controle ou de coorte prospectiva. Um primeiro desafio na elaboração de pesquisa de base populacional na Amazônia é a definição da população-alvo. Definir critérios de inclusão e exclusão esbarra em questões como: a falta de registro adequado de localidades na escala intramunicipal; a inadequação da classificação de áreas urbanas ou rurais; e a falta de sistema classificatório para identificar localidades com características mais ribeirinhas ou de terra firme ou áreas socialmente distintas, como remanescentes quilombolas. Essas bases geográficas não são consolidadas, resultando em inconsistências que afetam a qualidade dos estudos.

O tamanho populacional é outro desafio. O delineamento de estudos epidemiológicos tradicionais necessita de boas estimativas de população para embasar cálculos amostrais. Se a população for grande o suficiente para permitir um desenho amostral, a geração de inferências requer conhecer a população em risco. A falta de dados populacionais é comum nessa escala. Dados de setores censitários podem ser uma opção, mas, às vezes, eles não são harmonizados com bases territoriais identificadas socialmente ou ecologicamente. Outra fonte são dados da atenção primária, como da saúde da família ^6^. A mobilidade das pessoas também afeta os estudos populacionais, pois é frequente a mudança de moradia dentro do mesmo município, ou múltiplas moradias em áreas rurais e urbanas, e isso deve ser levado em conta na coleta de dados ^6^ ^,^ ^7^. A construção de uma moradia é relativamente fácil, principalmente nos pequenos municípios, e a unidade familiar varia bastante, com mudanças recorrentes na composição dos moradores das residências ^7^. Isso pode levar a perdas consideráveis do acompanhamento, sendo necessário delinear estratégias específicas para o estudo.

Em localidades com populações pequenas, pode ser necessário realizar censo e obter resultados apenas descritivos. Esses estudos fornecem conhecimento valioso sobre certas populações; por exemplo, povos indígenas de uma certa região ou uma pequena população ribeirinha com modo de vida tradicional ^9^ ^,^ ^10^.

A Amazônia abrange regiões metropolitanas no meio da floresta, núcleos urbanos, comunidades e vilas, áreas indígenas, quilombolas, fazendas, arranjos campesinos, seringais, garimpos, acampamentos de grandes projetos, assentamentos de reforma agrária e acampamentos sem-terra. A sazonalidade afeta a acessibilidade, com melhor acesso por estrada no período seco e melhor acesso fluvial no período chuvoso. Situações de conflito, desastre ambiental, comércio ilegal no território amazônico acrescentam mais uma camada de complexidade para a saúde das populações e sua acessibilidade. Essas heterogeneidades espaciais e temporais de acesso podem resultar em viés de observação, se a exposição, por exemplo, correlacionar com esses fatores espaço-temporais. Superar esses vieses requer investimento em infraestrutura de pesquisa e maior custo, inclusive de tempo. Pesquisas em localidades ribeirinhas tendem a necessitar de mais tempo de deslocamento por ter moradores mais distantes uns dos outros. Outro obstáculo a ser superado é a própria condição remota das populações de estudo, frequentemente em locais sem energia, em que o uso de aparelhos eletrônicos para a coleta de dados se torna difícil ou até mesmo impossível; essa condição é frequente em populações ribeirinhas ou rurais nas quais não há energia ou somente energia a gerador, de alto custo e baixa previsibilidade.

Por fim, cada uma dessas localidades têm suas peculiaridades quanto ao acesso, o que determina a estratégia de seleção dos participantes, a composição da equipe de pesquisa e os próprios objetivos da pesquisa.

Um outro desafio de estudos na Região Amazônica é a seleção de instrumentos que sejam válidos para a região, já que, em sua maioria, são validados em populações de ambientes e com condições de vida muito distintas. A diversidade cultural, que inclui a diversidade linguística, coloca a necessidade de validação em escala local mediante estudos qualitativos. Um exemplo é a adaptação de escalas psicométricas de insegurança alimentar em populações indígenas vulneráveis ^11^. Outro exemplo são os indicadores socioeconômicos. Para definir renda na Amazônia, é preciso considerar a importância do mercado informal. O trabalho assalariado nem sempre existe; a renda pode ser extremamente variável, diferindo a cada mês, semana ou dia. A determinação da posse de bens de consumo pode ser uma boa estratégia quando a renda nominal é incerta ou não quer ser declarada ^6^ ^,^ ^9^, além de permitir a construção de índices de riqueza capazes de proporcionar estratificação por renda. Contudo, deve-se considerar que, para algumas populações, a renda monetária não está associada ao seu modo vida e à sua reprodução social. Considerar uma variedade de indicadores para capturar as situações de privação oferece uma visão mais completa das realidades socioeconômicas das populações amazônidas. Estudos sobre pobreza baseados em perspectiva multidimensional apontam para a complexidade na avaliação do que é ser pobre na Amazônia e como os indicadores tradicionais podem mascarar as reais privações da população ^12^.

Por exemplo, em diversos locais, o material do piso e do domicílio, a presença de água encanada dentro ou fora da moradia ou a posse de barco a motor são as variáveis determinantes para a classificação econômica ^6^. Vários autores ^6^ ^,^ ^7^ ^,^ ^9^ citam como principais descritores das condições socioeconômicas de populações urbanas e rurais no interior da Amazônia: a presença ou não de eletricidade no domicílio; o tipo de piso; a presença de esgoto a céu aberto; o tipo de instalação sanitária (fossa ou assento com descarga); o destino do lixo (queimado ou enterrado); a origem ou tratamento da água de beber (água mineral, água de poço ou torneira filtrada, fervida ou clorada); e o uso de lenha como combustível para cozinhar. Nas últimas décadas, o recebimento de auxílios governamentais, em especial o Bolsa Família, provou ser um indicador para a estratificação socioeconômica da população amazônica ^7^ ^,^ ^13^.

Outro ponto é a necessidade de pensar formas alternativas de identificação de pessoas na ausência de registros formais, especialmente em áreas menos urbanizadas. Por exemplo, os registros nominais e de idade costumam variar bastante. Frequentemente, indivíduos informam sobrenomes diferentes dos registrados em cartório, tanto pela informalidade da população como por questões de escolaridade. São evidências da essencialidade de validação de instrumentos em contexto local.

## Conclusão

O entendimento das diferentes realidades que compõem esse todo só é adquirido com esse olhar detalhado para as particularidades amazônicas que os estudos de base populacional possibilitam. Devido à sua grande extensão e à heterogeneidade ambiental e de ocupação humana, entender a Amazônia e como ocorrem seus processos de saúde só é possível com grande multiplicidade de estudos. São esses estudos que avançarão para além do uso de bases secundárias que muitas vezes tem variáveis padronizadas e pensadas para o território nacional com limitações para contextos específicos e que podem contribuir para romper narrativas de invisibilização de populações e processos. Além disso, estudos qualitativos também podem ajudar a analisar e compreender processos de saúde-doença entre as populações amazônicas, em escalas diferentes dos estudos quantitativos.

Para alcançar esse objetivo, é fundamental aumentar o incentivo à pesquisa na Amazônia, estratégia que tem recebido maior ênfase governamental recentemente, especialmente estimulando a pesquisa nas instituições locais. Em segundo lugar, necessita de estudos bem conduzidos, com o envolvimento de pessoas com boa formação em pesquisa na Amazônia e que conheçam essas especificidades. Esse objetivo pode ser atingido a curto e médio prazo com o fortalecimento das pós-graduações sediadas em instituições amazônicas e pelo engajamento em redes de colaborações. Essas redes precisam ter caráter aditivo, que permita o engajamento das pessoas, e não restritivo (como estabelecer pré-requisitos numéricos para submissão de propostas de pesquisa). A longo prazo, entretanto, essa política deve ser fortalecida com a capacitação de recursos humanos nos mais diferentes níveis, iniciando-se no ensino básico, e disponível para todos, quer residam nas cidades, nas florestas, no litoral ou no campo. E, por fim, é crucial que sejam traçadas estratégias de longa duração, de no mínimo uma década ou mais.
